# Extensive Caseous Deposits in the Lungs of a Drill

**Published:** 1881-07

**Authors:** 


### VIII.—EXTENSIVE CASEOUS DEPOSITS IN THE LUNGS OF A DRILL.

A medium-sized drill (*Cynocephalus papusi*) was sent to the dead-house, with a history of having suffered for some weeks from a severe coughing, and an apparent asthmatic affection. The dyspnœa appeared to be distressing, and the animal died seemingly exhausted.

At the autopsy both lungs were found to be extensively involved in process of caseous degeneration. On the right side the upper and middle lobes were the chief seats of the disease. The affected lobes showed no remnant of healthy pulmonary tissue, but were completely transformed into a moderately solid, cheesy substance. Several border crumbs were found in the otherwise uniformly caseous deposit. The pleura covering this collection of cheesy material seemed unaltered. The middle lobe bulged at one point to such an extent as to have pushed the heart far over to the left side.

On the left side the upper lobe was congested, but otherwise healthy. The middle and lower lobes, however, were just as extensively involved by caseous transformation as the diseased lobes of the right side. Throughout both lungs numerous cavities, of various sizes, were scattered, and these showed their lining membranes, and contained a softer, more purulent matter. The spleen was considerably enlarged, and it, too, was the seat of severe caseous nodules. In the liver a few deposits of this kind were also seen. The remaining organs were pale, but otherwise healthy.

According to the history furnished of this case, the whole process must be supposed to have been rather an acute one. In the human being, such extensive cheesy degenerations are often the result of chronic catarrhal pneumonia, or chronic tuberculosis. It would be interesting to learn, by more extended observations, whether this disorder forms a variety of caseous phthisis peculiar to monkeys in confinement, and running an invariably rapid course.

